# The involvement of regulatory non-coding RNAs in sepsis: a systematic review

**DOI:** 10.1186/s13054-016-1555-3

**Published:** 2016-11-28

**Authors:** Jeffery Ho, Hung Chan, Sunny H. Wong, Maggie H. T. Wang, Jun Yu, Zhangang Xiao, Xiaodong Liu, Gordon Choi, Czarina C. H. Leung, Wai T. Wong, Zheng Li, Tony Gin, Matthew T. V. Chan, William K. K. Wu

**Affiliations:** 1 Department of Anesthesia and Intensive Care, The Chinese University of Hong Kong, Shatin, Hong Kong, Hong Kong, Special Administrative Region of China; 2 State Key Laboratory of Digestive Disease, LKS Institute of Health Sciences, The Chinese University of Hong Kong, Hong Kong, Hong Kong, Special Administrative Region of China; 3 Department of Medicine and Therapeutics, The Chinese University of Hong Kong, Shatin, Hong Kong, Hong Kong, Special Administrative Region of China; 4 The Jockey Club School of Public Health and Primary Care, The Chinese University of Hong Kong, Hong Kong, Hong Kong, Special Administrative Region of China; 5 Laboratory of Molecular Pharmacology, Department of Pharmacology, School of Pharmacy, Southwest Medical University, Luzhou, 646000 Sichuan, People’s Republic of China; 6 Department of Orthopedics Surgery Peking Union Medical College Hospital, Chinese Academy of Medical Sciences & Peking Union Medical College, Beijing, People’s Republic of China

**Keywords:** Sepsis, microRNA, lncRNA, circRNA, Biomarker, Inflammation, Shock

## Abstract

**Background:**

Sepsis coincides with altered gene expression in different tissues. Accumulating evidence has suggested that microRNAs, long non-coding RNAs, and circular RNAs are important molecules involved in the crosstalk with various pathways pertinent to innate immunity, mitochondrial functions, and apoptosis.

**Methods:**

We searched articles indexed in PubMed (MEDLINE), EMBASE and Europe PubMed Central databases using the Medical Subject Heading (MeSH) or Title/Abstract words (“microRNA”, “long non-coding RNA”, “circular RNA”, “sepsis” and/or “septic shock”) from inception to Sep 2016. Studies investigating the role of host-derived microRNA, long non-coding RNA, and circular RNA in the pathogenesis of and as biomarkers or therapeutics in sepsis were included. Data were extracted in terms of the role of non-coding RNAs in pathogenesis, and their applicability for use as biomarkers or therapeutics in sepsis. Two independent researchers assessed the quality of studies using a modified guideline from the Systematic Review Center for Laboratory animal Experimentation (SYRCLE), a tool based on the Cochrane Collaboration Risk of Bias tool.

**Results:**

Observational studies revealed dysregulation of non-coding RNAs in septic patients. Experimental studies confirmed their crosstalk with JNK/NF-κB and other cellular pathways pertinent to innate immunity, mitochondrial function, and apoptosis. Of the included studies, the SYRCLE scores ranged from 3 to 7 (average score of 4.55). This suggests a moderate risk of bias. Of the 10 articles investigating non-coding RNAs as biomarkers, none of them included a validation cohort. Selective reporting of sensitivity, specificity, and receiver operating curve was common.

**Conclusions:**

Although non-coding RNAs appear to be good candidates as biomarkers and therapeutics for sepsis, their differential expression across tissues complicated the process. Further investigation on organ-specific delivery of these regulatory molecules may be useful.

**Electronic supplementary material:**

The online version of this article (doi:10.1186/s13054-016-1555-3) contains supplementary material, which is available to authorized users.

## Background

Sepsis is defined as the presence of a life-threatening organ dysfunction as a result of altered systemic host response to an infection [1, 2]. This leads to multiple organ failure and superimposed secondary infections. The in-hospital mortality may reach 40% in the presence of septic shock [2, 3]. Recently, genome-wide expression analysis of the critically ill revealed more than 80% of the essential genetic elements were altered [4].

A class of non-coding RNAs, comprising microRNAs (miRNAs), long non-coding RNAs (lncRNAs), and circular RNAs (circRNAs), are increasingly being recognized as regulators of various signaling pathways and are thus known as regulatory RNAs. These molecules play important roles in biological processes, including innate immunity, mitochondrial functions, and apoptosis [5–9].

miRNAs are RNA molecules of 21 to 25 nucleotides in length synthesized in all healthy and diseased cells. By binding to complementary sequences in the 3’ untranslated regions of target mRNAs, miRNAs regulate a range of genes post-transcriptionally [10]. These regulatory polynucleotides play dual roles, either protective or detrimental, in cancers, neurodegenerative diseases, and immune-related diseases [11]. Notably, miRNAs are essential for the production of proinflammatory tumor necrosis factor (TNF)-α and interleukin (IL)-1β via p38 mitogen-activated protein kinase (MAPK) and MAPK phosphatase 1 (MKP-1) pathways [6–9, 12–14]. In case-control studies, differential expression of miRNAs was detected in patients with sepsis compared to controls, suggesting that miRNAs may be used as biomarkers for diagnosis and prognostic stratification or as therapeutic targets [8, 12, 15–21].

lncRNAs comprise more than 200 nucleotides, representing another group of transcripts. The mechanisms of lncRNAs in health and disease have been comprehensively reviewed [22, 23]. Recently, several in*-*vitro studies have documented the differential expression of lncRNAs in human tubular epithelial cells, monocytes, and cardiomyocytes after exposure to the plasma of septic patients or lipopolysaccharide (LPS) [24–26]. The role of lncRNAs in sepsis remains largely unknown. Massive screening of lncRNAs in human umbilical vein endothelial cells revealed that LPS treatment altered the expression of these non-coding RNAs by 28- to 70-fold [27]. Sporadic studies indicate that these changes might modulate inflammatory response. For instance, a lncRNA designated lnc-IL7R interacts with the human IL-7 receptor α subunit gene and hence alleviates the LPS-induced proinflammatory response [28]. In a murine sepsis model, lncRNA-HOTAIR appeared to modulate TNF-α production in cardiomyocytes via the nuclear factor (NF)-κB pathway [25].

circRNAs have been recognized as a distinct entity of non-coding regulatory RNAs fairly recently [29]. The circular structure stabilizes these molecules, favoring their use as biomarkers. Although our understanding of this new molecular member in sepsis remains sparse, experimental knockdown of a circRNA, RasGEF1B, deciphers the complex interaction of multiple cellular pathways in sepsis [30].

In this systematic review, we discuss the new paradigms of regulatory non-coding RNAs in the pathogenesis of sepsis and their potential as biomarkers and therapeutic targets.

## Methods

### Searching strategy and selection of studies

We searched articles indexed in PubMed (MEDLINE), EMBASE and Europe PubMed Central databases using Medical Subject Heading (MeSH) or Title/Abstract words (“microRNA or miRNA or lncRNA or circRNA” and “sepsis or septic shock”) from inception up to 30 Sep 2016. There were no limitations imposed on language or type of study. We included original research articles in which the role of host-derived regulatory RNAs (miRNA, lncRNA, or circRNA) in sepsis was examined in relation to disease pathogenesis, diagnosis, prognosis, and treatment. Investigation on exogenous regulatory RNAs or non-original research articles such as review articles, conference proceedings, editorials, and book chapters were excluded. Titles and abstracts were independently screened for relevancy by two authors. Disagreement was resolved by consensus or consultation with senior authors.

### Data extraction and study quality assessment

Data were extracted in terms of the role of non-coding RNAs (i.e., non-coding RNA species investigated, laboratory detection methods, and cellular pathways) and their use as biomarkers or therapeutic agents. The following data were abstracted: (1) first author and year of publication; (2) type of study; (3) non-coding RNA species investigated; (4) methods used to detect the corresponding non-coding RNA; (5) number of replicates/specimens (in vitro and in vivo studies) or patients (clinical studies); (6) cellular pathways involved; and (7) major conclusions. Two researchers independently performed the data extraction and evaluated the quality of the included studies using a modified guideline from the Systematic Review Center for Laboratory Animal Experimentation (SYRCLE), a tool based on the Cochrane Collaboration Risk of Bias tool [31]. One item concerning random housing of animals was removed in the modified version. This tool contains nine items assessing selection bias, performance bias, detection bias, attrition bias, and reporting bias. These factors are common amongst in vitro, in vivo, and human studies. The higher the SYRCLE score was, the better the quality of the study would be. The maximum achievable score is 9.

## Results

**Fig. 1:**
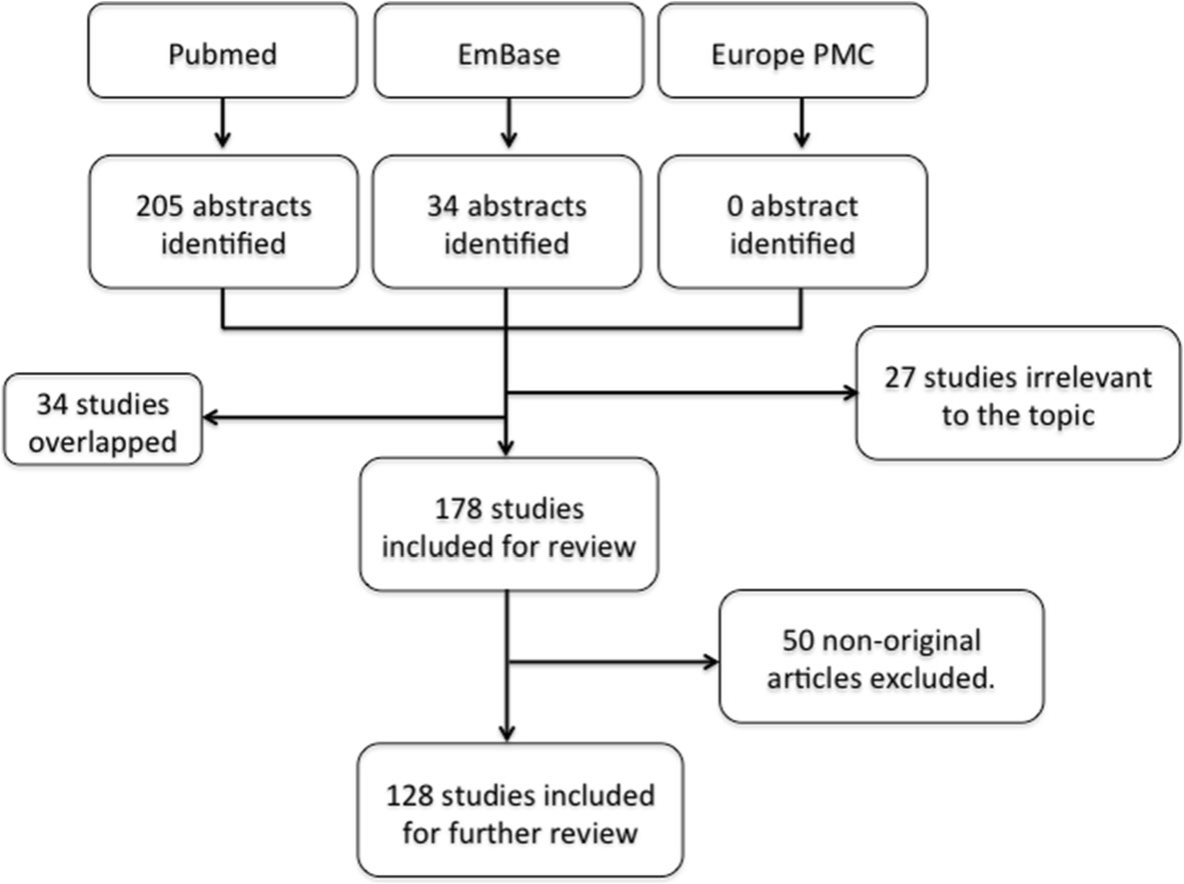
Selection of studies. *PMC* PubMed Central

A total of 239 papers were found based on the search criteria, in which 128 original studies investigating miRNAs, lncRNAs, or circRNAs in sepsis were included. Of these, eight articles examined the role of lncRNAs or circRNAs, whereas the remaining investigated miRNAs. The papers excluded were either not original articles, or not directly related to sepsis, or had lack of evidence of deregulation of the studied miRNA/lncRNAs/circRNAs in sepsis (Fig. 1). Two authors independently searched the literature database and agreed with the data abstracted as summarized in Additional file 1 (Table S1). Of the 128 included studies, 24, 28, and 20 were purely in vitro, in vivo, or human studies, respectively. The remaining employed multiple models (i.e., a combination of in vitro, in vivo, or human studies). The SYRCLE scores ranged from 3 to 7, with an average score of 4.55.

In general, studies of non-coding RNAs in sepsis focus on immunological dysregulation and evaluation of these as biomarkers. Other active research areas include the impact of their alteration on endothelial dysfunction, organ failure, and evaluation as therapeutic agents. These are summarized in Table 1. An altered expression of non-coding RNAs involves multiple cellular populations and signaling pathways leading to changes in immune response, hormonal imbalance, metabolic and mitochondrial dysfunction, epithelial integrity, and coagulation-defects [1, 3, 31–36].

**Table 1:** Summary of search results

Themes	No. of studies^a^	Key cellular pathways involved
Altered miRNA expression	28	Integrin signaling, leukocyte extravasation, apoptosis
Immune dysfunction	31	TNF-α/TLR/NF-κB
Endothelial dysfunction	7	MAPK/EGR, AP1/ NF-κB
Cardiopulmonary impairment	20	JNK PPARγ
Defects in other major organs	12	cAMP, Hxm1
Biomarkers (in vitro, in vivo, and clinical evidence)	28	Various
Therapeutic agents	17	Various

^a^The total number of studies is not equal to 128 due to multiple themes addressed by the same article

*AP1* activator protein 1, *cAMP* cyclic adenosine monophosphate, *EGR* early growth response, *JNK* cJun NH2-terminal kinase, *MAPK* mitogen-activated protein kinase, *miRNA* microRNA, *NF* nuclear factor, *PPAR* peroxisome proliferator-activated receptor, *TLR* Toll-like receptor, *TNF* tumor necrosis factor

**Fig. 2:**
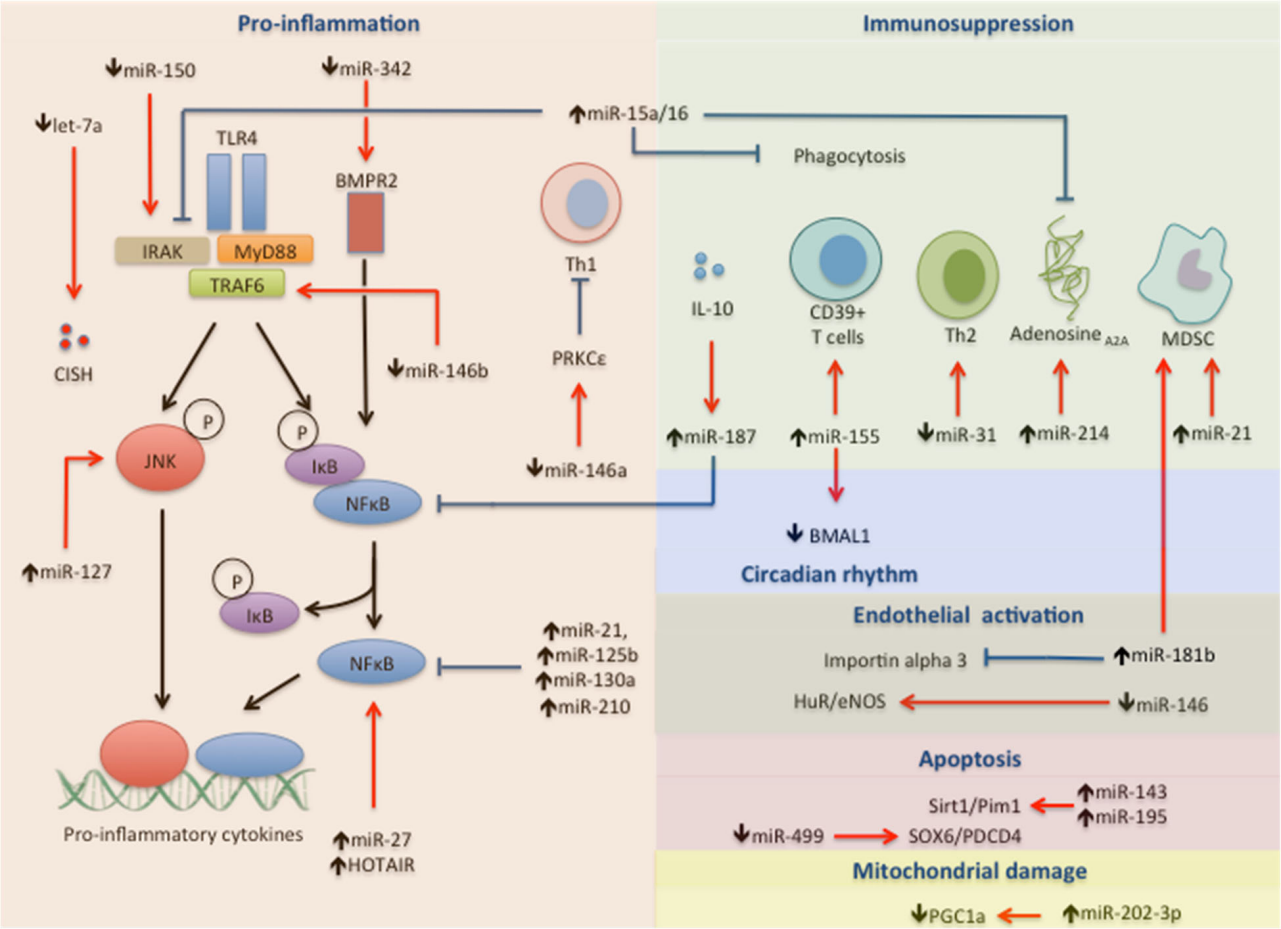
Role of microRNA (*miRNA*) and long non-coding RNA (*lncRNA*) in sepsis. HuR, Human antigen R; eNOS, Endothelial nitric oxide synthase; MDSC, Myeloid derived suppressor cell; CISH, Cytokine-inducible SH2-containing protein; JNK, c-Jun N-terminal kinases (JNK); IRAK, Interleukin-1 receptor-associated kinase; MyD88, Myeloid differentiation primary response gene 88; TRAF6, TNF receptor-associated factor 6; BMPR2, Bone morphogenetic protein receptor type II; IkB, Inhibitor of Kappa B; NFkB, Nuclear factor kappa B; Sirt1, Sirtulin 1; Pim1, Proto-oncogene serine/threonine-protein kinase; SOX6, Sex-determining region Y box 6; PDCD4, Programmed cell death 4; BMAL1, Brain and muscle ARNT-like 1; PRKC, Primary rat kidney cell; PGC1a, PPAR gamma co-activator 1A

Changes in miRNA expression are detectable after exposure of cells, animals, or healthy human volunteers to sublethal concentration of LPS. Some of the miRNAs (e.g., miR-155, miR-143) are upregulated while many others (e.g. miR-125b, miR-146b, miR-150, miR-340, let7g) are downregulated [12, 37–48]. The intricate crosstalk between miRNAs and various cellular pathways is depicted in Fig. 2.

Expression of non-coding RNAs differs in response to the specific microbial moiety encountered. For instance, the expression level of let-7a in leukocytes was reduced in healthy humans after exposure to LPS but not after exposure to lipoteichoic acid or double-stranded RNA [38]. Similarly, the expression of miR-150 was reduced significantly after exposure to LPS but was elevated when challenged by lipoteichoic acid and polyinosinic-polycytidylic acid double-stranded RNA [13].

Of the 22 subpopulations included in the 10 articles evaluating non-coding RNAs as biomarkers, none of them included an external validation cohort. Selective reporting of sensitivity, specificity, and receiver operating curve was common. Reporting of sensitivity and specificity was absent in 13 subpopulations. Two authors reported only an area under the curve (AUC) value without a confidence interval. The study population and the non-coding RNAs investigated were heterogeneous, precluding the performance of meta-analysis. The sensitivity of miRNA biomarkers ranges from 38 to 80%, whereas specificity ranged from 71.4 to 100%. The predictability of miRNA markers is summarized in Table 2.

**Table 2:** Use of microRNAs as diagnostic or prognostic markers

						95% CI		
	No. of subjects (*n*)	Specimen	Sensitivity (%)	Specificity (%)	AUC	Lower bound	Upper bound	Reference
Diagnostic markers								
miR-146a	SIRS (14) vs sepsis (14)	Plasma	60	87.5	0.813	0.608	1.017	[51]
miR-15a	Healthy people (32) vs severe sepsis (62)	Plasma	n/a	n/a	0.7	0.57	0.84	[15]
miR-27a	Healthy people (32) vs severe sepsis (62)	Plasma	n/a	n/a	0.66	0.52	0.8	[15]
miR-34a	Healthy people (32) vs severe sepsis (62)	Plasma	n/a	n/a	0.67	0.53	0.8	[15]
miR-15a + 27a + 34a	Healthy people (32) vs severe sepsis (62)	Plasma	n/a	n/a	0.78	0.66	0.9	[15]
miR-150	Healthy (21) vs SIRS (22) vs sepsis (23)	Whole blood	72.7	85.7	0.83	n/a	n/a	[16]
miR4772-5p-iso	Healthy (21) vs SIRS (22) vs sepsis (23)	Whole blood	68.2	71.4	0.76	n/a	n/a	[16]
miR-25	SIRS (30) vs sepsis (70)	Whole blood	n/a	n/a	0.806	0.701	0.912	[17]
miR-15a	SIRS (32) vs sepsis (166)	Serum	68.3	94.4	0.858	0.8	0.916	[18]
miR-16	SIRS (32) vs sepsis (166)	Serum	n/a	n/a	0.55	0.455	0.644	[18]
miR-223	Normal control (24) vs sepsis (166)	Whole blood	38.21	83.72	0.608	0.519	0.697	[19]
miR-499-5p	Normal control (24) vs sepsis (166)	Whole blood	68.29	65.12	0.686	0.592	0.779	[19]
miR-126	SIRS (30) vs sepsis (50)	Serum	n/a	n/a	0.607	0.448	0.766	[21]
miR-146a	SIRS (30) vs sepsis (50)	Serum	63.3	100	0.804	0.679	0.928	[21]
miR-223	SIRS (30) vs sepsis (50)	Serum	80	100	0.858	0.748	0.968	[21]
miR-15a	Neonates with RTI (41) vs septic neonates (46)	Serum	n/a	n/a	0.854	n/a	n/a	[53]
miR-15b	Neonates with RTI (41) vs septic neonates (46)	Serum	n/a	n/a	0.629	n/a	n/a	[53]
miR-16	Neonates with RTI (41) vs septic neonates (46)	Serum	n/a	n/a	0.869	n/a	n/a	[53]
miR-223	Neonates with RTI (41) vs septic neonates (46)	Serum	n/a	n/a	0.632	n/a	n/a	[53]
Change of miRNA associated with poor prognosis								
↓miR-25	SIRS (30) vs sepsis (70)	Whole blood	n/a	n/a	0.756	0.569	0.833	[17]
↑miR-155	Healthy people (30) vs septic patients (60)	Whole blood	n/a	n/a	0.763	0.626	0.901	[71]
↓miR-574-5p	Sepsis survivors (12) vs sepsis nonsurvivors (12)	Serum	54.55	96.15	0.736	0.646	0.827	[127]

*CI* confidence interval, *miRNA* microRNA, *n/a* not available, *RTI* respiratory tract infection, *SIRS* systemic inflammatory response syndrome

## Discussion

### Pathogenesis

This literature review indicates that the pattern of non-coding RNA expression differs substantially upon encountering various microbial moieties [38, 41]. Analysis of peripheral blood by quantitative RT-PCR and miRNA microarrays has been widely used for expression profiling of miRNA in septic patients [15, 16, 49, 50]. Notably, several miRNA species, including miR-126, miR-21, miR-16, and miR-27a, increased more than 30-fold in sepsis [15]. Single-candidate miRNA studies have established the association of miR-146a, miR-25, and miR-15a/16 with sepsis [17, 51–54]. Further stratification of the systemic inflammatory response syndrome (SIRS) from sepsis revealed differential miRNA deregulation [16]. These results suggest that miRNA expression may be pathogen-specific and that its pattern could be used as biomarkers or therapeutic targets.

#### Immunological changes

miRNAs and lncRNAs are involved in both proinflammatory and anti-inflammatory responses in sepsis [55–58]. Notably, the majority of lncRNAs responsive to LPS stimulus contain one or more binding sites for known inflammatory mediators such as p65, IRF3, JunB, and cJun [58]. Exposure of cell lines and animal models to LPS is a popular method for investigating their roles in inflammation. In an LPS model of murine sepsis, an increased expression of miR-15a/16 reduced the phagocytic activity of macrophages and increased mitochondrial oxidative stress, resulting in a proinflammatory phenotype [59, 60]. Overexpression of miR-15a/16 in the LPS-treated murine macrophage RAW264.7 downregulated the expression of Toll-like receptor (TLR)4 and IL-1 receptor-associated kinase 1 (IRAK1), contributing to immunosuppressive phenotypes [53, 61]. Similarly, expression of miR-205-5b alleviates the expression of high mobility group box 1 (HMGB1) [62].

The production of proinflammatory TNF-α is finely controlled at both the transcriptional and translational levels by miRNAs. Upregulation of miR-181 enhances TNF-α mRNA degradation [60]. In a THP-1 human promonocytic cell model, miR-146a increased in a time-dependent manner on LPS/TLR4 stimulation, suppressing the production of proinflammatory cytokines in a feed-forward loop [63, 64].

Cytokine homeostasis can also be achieved in a negative feedback manner. Upregulation of miR-146a inhibits IRAK-1 and p-IKBa in THP-1 cells exposed to *S. typhimurium* [65]. Binding of NF-κB to DICER increased the levels of mature miR-125b in hepatocytes, suppressing TNF-α expression [66]. In CD14^+^ neonatal monocytes, enforced expression of miR-125b suppresses translation of TNF-α [67]. Silencing of CD14 by small interfering (si)RNA abolishes the production of TNF-α and IL-6 [68].

As sepsis progresses, the immune system is reprogrammed into a stage characterized by persistent inflammation and immunosuppression [69, 70]. These are mediated in part by miRNAs, which promote immune cell polarization, suppress proinflammatory cytokines, and control leukocyte apoptosis [71–74]. For instance, the expression of miR21 and miR-181b in myeloid -derived suppressor cells in septic mice precludes the differentiation of macrophages and dendritic cells [72, 75–77]. Given the extensive crosstalk between miRNAs and other cellular pathways, inflammatory responses can be modulated by interfering upstream or downstream mediators. By targeting Bmal1, NF-κB-p65/RelA phosphorylation was inhibited by miR-155 [78, 79]. Interestingly, transcription of primary miR-155 and other three miRNAs (miR-455, miR-125a, and miR-146) is dependent on NF-κB [80]. The expression of NF-κB and its interaction with miRNAs has been demonstrated in trauma patients with sepsis [81]. Upregulation of miR-19a in patients with sepsis or SIRS correlates with the extent of systemic inflammation [82]. Experimental silencing of miRNAs has further confirmed the importance of these regulatory nucleotides in limiting inflammation in sepsis. Transfection of anti-miR-210 into the murine macrophage RAW264.7 and human HEK293 cells enhanced LPS-induced production of IL-6, TNF-α, and inducible nitric oxide synthase (iNOS) [83, 84].

Observational studies have demonstrated an association between sepsis-induced coagulopathy and miRNA expression [85, 86]. Compared with severe sepsis patients with normal platelet counts, the expression of miR-130a in peripheral blood monocytic cells was significantly lower in septic patients with thrombocytopenia [86]. Longitudinal samples of sepsis patients revealed a sustainable increase of miR-122 up to 14 days after admission to the intensive care unit and showed a strong correlation with antithrombin III (*R* = 0.913, *p* < 0.001) [87]. To determine direct or indirect effects of miRNAs on coagulation, further mechanistic studies are required to identify crosstalk, if any, between cytokines, thrombocyte synthesis/apoptosis, and deregulation of miRNAs.

#### Endothelial dysfunction

Sepsis-induced endothelial activation and injury is mediated in part by the Slit2-Robo4 pathway [88]. Downregulation of Slit2 reduced the expression of miR-218, modulating endothelial inflammation [88]. A disintegrin and metalloproteinase (ADAM)15 is another mediator responsible for increased endothelial permeability. An in-silico analysis of human vascular endothelial cells revealed that miR-147b degrades ADAM15 mRNA. The endothelial protective function of miR-147b was further confirmed by in vitro experiments of overexpression and co-incubation with miR-147b antagomir (a miRNA inhibitor) [89]. In response to proinflammatory cytokines, miR-146a/b expression in endothelial cells is also increased [90]. These miRNAs target NF-κB, activator protein-1 (AP-1), and MAPK/Egr-1 pathways and, in turn, abolish the production of proinflammatory cytokines in a negative-feedback loop. Knocking out miR-146a in mice aggravates the expression of VCAM-1 in the endothelium. These collectively suggest that miRNAs prevent endothelial activation, which may otherwise be aggravated by proinflammatory cytokines in sepsis [91–93].

#### Cardiopulmonary impairment

The lung accounts for more than 45% of the primary sites of infection in sepsis patients [79]. Development of acute respiratory distress syndrome is one of the serious complications seen in sepsis patients; the prevalence ranges from 6 to 16% [94–96]. Amongst those who developed severe sepsis, the prevalence of cardiovascular and respiratory failure increased up to 90% [97].

In the context of sepsis-associated pulmonary injury, miRNAs interfere with JNK/PPARγ and cholinergic pathways which, in turn, contribute to pulmonary inflammation or inflammatory resolution [98–103]. Exposure of rats to LPS increased the production of TNF-α and IL-1β in the myocardium accompanied by upregulation of miR-194-3p, miR-344a-3p, miR-465-3p, miR-501-5p, miR-3596c, miR-185-3p, and miR-877 [104]. In vitro studies showed that the increased expression of miR-127 de-represses Bcl6/Dusp1, which in turn activates JNK and promotes macrophage polarization toward the M1 phenotype [100, 105]. Intratracheal administration of miR-127 in mice has confirmed M1 skewing and exaggerated pulmonary edema and infiltration [100]. Peculiarly, the level of miR-127 decreased transiently during the very early stages of sepsis in an attempt to minimize pulmonary inflammation. The mechanisms leading to subsequent sustainable de-repression remain unknown.

Sepsis-induced cardiac dysfunction is characterized by impaired myocardial contractility and reduced ejection fraction [106]. Increasing evidence has suggested the role of lncRNAs and miRNAs in these processes [24, 25, 107–110]. In myocardial cells, a lncRNA, HOTAIR, was induced after exposure to LPS. This correlates with increased TNF-α production and NF-κB p65 phosphorylation [25]. Investigation of neonatal rat cardiomyocytes demonstrated that LPS inhibited the expression of miR-499, which in turn de-repressed SOX6 and PCDC4 leading to cardiomyocyte apoptosis through activation of the Bcl-2 family apoptotic pathway [111].

#### Implications on other major organs

A global observational study involving 14,573 severe sepsis patients from 37 countries revealed that a considerable proportion of them developed hepatic (20%) and renal impairment (40%) [97].

Ample evidence has indicated a change in miRNAs/lncRNAs in the liver [112, 113], kidneys [114, 115], and skeletal muscles [116, 117] in sepsis, associated with organ failure. In a rat model of sepsis, the upregulation of miR-142-3 was detected by 18 h after cecal ligation and puncture (CLP). This change in expression level reduced adenylyl cyclase 9 expression in liver macrophages, which may prevent macrophages from resolving the proinflammatory response in a cyclic adenosine monophosphate (cAMP)-dependent manner [112]. Experimental knockdown of miR-21 in LPS-septic mice resulted in upregulation of programmed cell death protein 4, increased apoptosis, and exacerbated LPS-induced kidney injury [114]. Sequencing of RNA extracted from human proximal tubular epithelial cells after exposure to plasma from septic humans with acute kidney injury revealed significantly increased expression of linc-ATP13A4-8 as compared with exposure to those from septic patients without kidney involvement [26]. Urosepsis caused by *Candida spp.*, although infrequent, is responsible for high mortality and severe kidney injury. Intraperitoneal injection of *Candida albicans* into C57BL/6 mice revealed an impaired renal glomerular filtration rate accompanied by a significant reduction in miR-204/211, leading to upregulation of a heme oxygenase, Hmx1 [115]. Administration of miR-204/211 mimics reduced the expression of Hmx1 and alleviated kidney injury. These results confirmed the protective role of miR-204/211 in maintaining kidney functions via Hmx1 in sepsis.

Limited studies have investigated the role of miRNAs in sepsis-induced myopathy [117, 118]. Clinical studies revealed that muscle-associated miRNAs are dysregulated in sepsis [118]. In a porcine sepsis model, significant upregulation of two miRNA species (miR-146-5p and miR-221-5p) was detected, suggesting the possible involvement of these miRNAs in muscle catabolism in sepsis [117].

### Biomarkers

While regulatory RNAs have been recognized for more than a decade, their use as biomarkers for sepsis diagnosis and prognostication has not been thoroughly investigated until recently (Table 1). Microarray analyses, next-generation sequencing, and quantitative RT-PCR are important tools in developing biomarkers [15–21, 50, 51, 53, 57, 71, 97, 107, 119–131]. To date, candidate regulatory RNAs are limited to miRNAs. No study has evaluated the feasibility of using lncRNAs or circRNAs as biomarkers in sepsis.

Investigations revealed that the expression level of miR-25, miR-143, miR-146a, miR-15a, miR-16, miR-126, miR-150, miR-223, and 472-5p-iso could differentiate SIRS from sepsis [16–21, 48–51, 107, 132]. However, an independent research group could not detect any difference in miR-223 expression between septic patients and healthy controls [110]. Recently, massive screening using Solexa sequencing has identified nine novel miRNAs which are correlated with sepsis mortality (AUC = 0.681–0.863) [50]. Of note, selective reporting of sensitivity, specificity, and the associated AUC value was common in several included studies. In addition, none of these studies used an external cohort to validate the biomarkers investigated. This reporting bias complicates the analysis of results between studies.

Other miRNAs have been investigated to predict complications associated with sepsis. For instance, miR-122 predicts the development of liver injury in septic patients [70, 126]. miR-574-5p and miR-155 may predict the development of septic shock, immunosuppression and respiratory failure [71, 127, 133, 134].

Clearly, genome-wide profiling of miRNA expression distinguished septic from nonseptic patients. However, prediction of the likelihood of a nonseptic patient developing sepsis may be more clinically relevant to reduce mortality and morbidity in critical care settings.

### Therapeutic targets

Although cell and animal models have demonstrated the use of miRNA modulators in combating sepsis, considerable challenges have to be overcome in order to successfully translate these into clinical use. Apparently, the expression of miRNAs is tissue-dependent, questioning the appropriateness of systemic delivery of antagomir or miRNA mimics, as has been commonly performed in animal models. Although targeted drug delivery may be an alternative, this is further complicated by the technology of the delivery and the heterogeneity of clinical manifestations among septic patients.

Recent animal studies have recognized that miRNAs are associated with medical interventions and septic complications [125–138]. In SPRET/Ei mice, glucocorticoid induced miR-511 upregulation, inhibiting the TNF receptor TNFR1 and, hence, reducing their sensitivity to TNF-α [135]. Similarly, administration of dexamethasone in a LPS-induced murine sepsis model downregulated the expression of miR-155 in the liver and alleviated proinflammatory cytokine production [116, 138]. Interestingly, miRNAs are also involved in cortisol nonresponsiveness, which may occur during the therapy. This resistance phenotype is partly related to altered expression of one of the cortisol receptor isoforms, glucocorticoid receptor α. In sepsis patients, this isoform is significantly downregulated by miR-124, which is increased three-fold upon exposure to glucocorticoid [137].

After exposure to LPS for a while, our body switches to tolerance mode, which avoids prolonged proinflammatory response. Silencing transcription and translation of acute inflammatory genes in vitro during LPS tolerance is mediated by various miRNAs [139]. Regulation of this tolerance status can be significantly disrupted by overexpression of miR-146a in cellular models of sepsis [140]. While sepsis-induced differential miRNA expression involves a diversity of miRNA species in multiple organs, modulation of miRNAs in endotoxin tolerance is seen predominantly in macrophages mediated by miR-146a and miR-155 [141, 142]. As in sepsis, in vitro studies suggested that the regulation of miRNA during tolerance involves the TLR-NFkB-cytokine pathways [143–145].

Experimental evidence suggests that alternative therapies in treating sepsis involve modulation of miRNAs. Predominantly, these miRNA species are linked to some, if not all, pathways in innate immunity in cellular [146, 147] and in animal models [148, 149]. One of the well-known anti-inflammatory dietary components is flavonoids. In vitro investigation of a flavonoid, apigenin, revealed that it suppresses LPS-induced miR-155 expression in macrophages, leading to upregulation of the anti-inflammatory regulators forkhead box O3a and MAD-related protein 2 [146]. The protective effect was further elaborated by a murine sepsis model in which an apigenin-rich diet considerably reduced the expression of miR-155 and TNF-α in the lungs [146]. Recent innovations in septic treatment include stem cell therapy. In this regard, in vivo mechanistic studies revealed that mesenchymal stem cells improved survival of CLP-induced septic mice by downregulating miR-143 [147, 150]. Microarray analyses revealed more than 1.5-fold differential expression of 77 miRNAs in septic mice treated with noncultured-derived mesenchymal cells [149]. This was accompanied by a reduced inflammatory response and apoptosis [149]. Elucidating the mechanisms using animal models of sepsis in relation to a 20-HETE analog, N-(20-hydroxyeicosa-5Z,14Z-dienoyl glycine, revealed the involvement of miR-150, miR-223, and miR-297 [148]. Further in vitro studies revealed that these miRNAs were downregulated leading to suppression of the MyB88/NF-κB pathway [151].

Evaluation of the feasibility of miRNA as septic therapy predominantly employs two approaches: use of antagomir or miRNA mimics. Among all miRNAs, miR-146a is the most comprehensively studied candidate. By targeting IRAK1 and TRAF6, miR-146a attenuates cardiac dysfunction in septic mice [152]. Delivering miR-146a agomir by in vivo jetPEITM instillation into airways of septic mice inhibited proinflammatory cytokine production and alleviated lung tissue injury [153]. An independent group of researchers revealed that this miRNA additionally interferes in vitro with Th1 cell differentiation of human CD4^+^ T lymphocytes via PRKC [154]. Other miRNAs, including miR-124, miR-142-3p, and miR-195, have also been demonstrated to be useful in preventing hyperinflammation, apoptosis, and multiple organ injury in murine sepsis models [8, 140, 155, 156]. Similarly, indirect induction of miR-126 expression in vitro by CTEC-0214, a stromal cell-derived factor 1 alpha analog, preserved endothelial cell barrier integrity and attenuated pulmonary vascular leak [157].

## Conclusion

In conclusion, regulatory non-coding RNAs are potential candidates as biomarkers and therapeutics for sepsis. Given organ-specific differentiation of these regulatory non-coding RNAs in addition to the pathological heterogeneity of patients with sepsis, future research is warranted to elucidate the temporal dynamics and cellular origins of regulatory RNAs. Development of organ-specific delivery of non-coding RNA mediators may be a promising approach.

## Additional file

Additional file 1: Table S1. a Summary of the included studies. b Quality assessment of the included studies according to SYRCLE score. (DOCX 73 kb)

## Abbreviations

ADAM: A disintegrin and metalloproteinase
AP-1: Activator protein-1
AUC: Area under the curve
circRNA: Circular RNA
CLP: Cecal ligation and puncture
HMGB1: High mobility group box 1
IL: Interleukin
iNOS: Inducible nitric oxide synthase
IRAK1: Interleukin-1 receptor-associated kinase 1
lncRNA: Long non-coding RNA
LPS: Lipopolysaccharide
MAPK: Mitogen-activated protein kinase
miRNA: MicroRNA
MKP-1: MAPK phosphatase 1
NF: Nuclear factor
SIRS: systemic inflammatory response syndrome
SYRCLE: Systematic Review Center for Laboratory Animal Experimentation
TLR: Toll-like receptor
TNF: Tumor necrosis factor
