# Supplementary material for: The involvement of regulatory non-coding RNAs in sepsis: a systematic review
Source: Crit Care. 2016 Nov 28;20:383. doi: 10.1186/s13054-016-1555-3 (PMC5125038; doi:10.1186/s13054-016-1555-3)
Supplement: Additional file 1: Table S1. — a Summary of the included studies. b Quality assessment of the included studies according to SYRCLE score. (DOCX 73 kb) [file 13054_2016_1555_MOESM1_ESM.docx]

**Table S1A. Summary of the included studies.**

| First author, Year [Reference] | Setting | Non-coding RNA investigated | Methods | Number of replicates/ subjects | Cellular pathways involved | Major conclusion | SYRCLE score |
| --- | --- | --- | --- | --- | --- | --- | --- |
| Brudecki, 2013 [6] | In vitro | miR146a | RT-PCR | 3 | p38 MAPK | miR146a limits inflammation | 3 |
| Brudecki, 2013 [7] | In vitro | miR146a | qPCR | 3 | TLR4, p38MAPK | miR146a modulated the translation of proinflammatory genes during the acute inflammatory response | 3 |
| Liu, 2014 [24] | In vitro | lincRNAs | RNA-seq,qRT-PCR | 6 | - | LPS-mediated lincRNAs were correlated to cardiometabolic traits | 3 |
| Lin, 2015 [26] | In vitro | MIR210HG, linc-ATP13A4-8, and linc-KIAA1737-2 | RAN-seq | N/A | - | The lncRNA landscape is associated with hypoxic and inflammatory stress | 3 |
| Liu, 2014 [35] | In vitro | miR-294 | RT-PCR | 3 | - | miR-294 was related to inflammatory response by reducing IL-6 secretion in LPS-induced RAW264.7 | 4 |
| Rossato, 2012 [43] | In vitro | miR-187 | ChIP, RT-PCR | 4 to 5 | - | miR-187 controlled cytokine expression in anti-inflammatory process | 5 |
| Heyn, 2012 [61] | In vitro | miR-214,miR-15,miR-16 | qRT-PCR | 12 | - | miRNA expression levels can be used as marker to distinguish the risk for severe inflammation | 3 |
| El Gazzar, 2011 [63] | In vitro | miR-146a | IP, ChIP,PCR | 3 | - | miR-146a contributes to siene acute proinflammatory genes | 3 |
| Quinn, 2013 [65] | In vitro | miR-146a | RT-PCR | 3 | TLR2 | miR-146a triggers tolerance in BLP-stimulated cells during sepsis | 3 |
| Du, 2014 [68] | In vitro | 199a-3p, miR-199a-5p, and miR-21-5p | qRT-PCR | 3 | - | miRNAs are associated with immune response, inflammatory response and cytokine production | 3 |
| Wang, 2014 [76] | In vitro | miR-30a | ELISA, RT-PCR | 3 | - | miR-30a limits the LPS-binding proteins in human monocytes | 3 |
| Monk, 2010 [80] | In vitro | miR-455, miR-125, miR-146 and miR-155 | Microarry, qRT-PCR | 4 | NF-κB, p38 MAPK | microRNAs adjust the macrophage functions | 3 |
| Song, 2015 [84] | In vitro | miR-29a | ELISA, RT-PCR | 3 | STAT3 | miR-29a limits STAT3 in human monocytes during sepsis | 3 |
| Chatterjee, 2014 [89] | In vitro | miR-147b | Immunofluorescence, flow cytometry | 3 | - | miR-147b regulates endothelial barrier function during inflammation and sepsis | 3 |
| Wu, 2015 [92] | In vitro | miR-23b | qPCR | N/A | - | miR-23b limits inflammatory factors | 3 |
| Liu, 2015 [99] | In vitro | miR-132 | qRT-PCR, ELISA | 3 | - | miR-132 has anti-inflammatory effect | 3 |
| Adyshev, 2013 [101] | In vitro | has-miR-374a,hsamiR-374b, hsa-miR-520c-3p, and hsa-miR-1290 | qRT-PCR | 3 | - | miRNAs are involved in modulation of inflammatory lung injury | 3 |
| Jia, 2016 [111] | In vitro | miR-499 | qRT-PCR | 3 | - | miR-499 has protective effect in cardiomyocytes against LPS-induced apoptosis | 3 |
| Shukla, 2015 [151] | In vitro | miR-30c-2-3p | RT-PCR | 3 | NF-κB | miR-30c-2-3p inhibits cell cycle progression in breast cance through negatively modulation of NF-κB signaling | 3 |
| Singh, 2016 [27] | In vitro | IncRNAs (AL132709.5 , CTC-459I6.1 ) | Microarray | 3 |  | IncRNA is involved in LPS-mediated changes in the vascular pathology of sepsis | 3 |
| Zhao, 2016 [156] | In vitro | MALAT1 | qPCR, ChIP | 2 | NF-κB | MALAT1 regulates innate immune responses | 3 |
| Cui, 2014 [28] | In vitro | lnc-IL7R | RT-PCR, ChIP | 3 |  | lnc-IL7R regulates inflammatory responses | 3 |
| Nahid, 2009 [141] | In vitro | miR-146a | qRT-PCR | 3 | TLR4 | miR-146a is important in endotoxin tolerance | 3 |
| Nahid, 2015 [144] | In vitro | miR-146a | qRT-PCR | 3 | LPS-TLR4 | miR-146a is important in IL-1b tolerance | 3 |
| Zheng, 2016 [8] | In vivo | miR195 | ELISA | 3 to 6 | - | inhibition of miR-195 improved the mortality of spesis and the mechanism was related to the activation of Bcl-2,sirt1 and Pim-1 | 5 |
| Kanann, 2012 [33] | In vivo | miRNA | RT-PCR | 3 | - | mIRNA limited the inflammatory cytokines | 4 |
| Wu, 2013 [39] | In vivo | miR-16, miR-17, miR-20a, miR-20b, miR-26a, miR-26b, miR-106a, miR-106b, miR-195, and miR-451 | Microarray | 6 | - | Ago2 complexes and exosomes contributed to the stability of miRNAs | 4 |
| Hsieh, 2012 [40] | In vivo | let-7d, miR-15b, miR-16, miR-25, miR-92a, miR-103, miR-107 and miR-451 | RT-CPR | 6 | - | whole blood-derived miRNAs can be biomarkers for LPS exposure | 4 |
| Barnett, 2016 [42] | In vivo | miR-21 | ELISAs | 8 | NF-κB | miR-21 modulated the macrophage in anti-inflammatory effects | 4 |
| Moore, 2013 [49] | In vivo | miR-182,-199a-5p, -203, -211, -222, -29b | qPCR | 6 | - | adjustment of miRNAs had the cytoprotective effects during sepsis | 6 |
| Wang, 2012 [55] | In vivo | let-7g, miR-101b, miR-181b, miR-455 | qRT-PCR | 8 | - | miRNAs were involved in the innate immune response | 4 |
| McClure, 2016 [73] | In vivo | miR-21, miR-181b | ELISA,Northern blots | 3 | - | regulation of miR-21,miR-181b is involved in spesis-associated immunosuppression | 4 |
| Piccinini, 2012 [79] | In vivo | miR-155 | ELISA, qRT-PCR | 6 | TLR4 | miR-155 is involved in the synthesis of anti-inflammatory cytokines | 5 |
| Sun, 2014 [103] | In vivo | miRNA-16 | Immunoprecipitation, flow cytometry | 3 | STAT, NF-κB | miR-16 contributes neutrophil proinflammatory cytokine production | 5 |
| Ding, 2015 [104] | In vivo | miR-194-3p, miR-344a-3p, miR-465-3p, miR-501-5p, miR-3596c, miR-185-3p, and miR-877 | RT-PCR | 20 | - | miRNAs are involved in endotoxin-induced myocardial injury | 4 |
| Wang, 2014 [108] | In vivo | miR-223 | miRNA array, qRT-PCR | 4 to 10 | - | miR-223 triggers the augmentation of sepsis-induced inflammation, myocardial dysfunction and mortality | 5 |
| Wang, 2016 [110] | In vivo | miR-21-3p | qRT-PCR,microarry | 6 | - | miR-21-3p contributes to the sepsis-related cardiac dysfunction | 5 |
| Risoe, 2011 [112] | In vivo | miR 142-3p | qPCR | 24 | - | miR142-3p induced AC9 reduction and resolved the proinflammatory responses | 5 |
| Wang, 2012 [113] | In vivo | miR-155 | ELISA, RT-PCR | 120 | - | miR-155 plays a critcial postion in liver injury during sepsis | 6 |
| Jia, 2015 [114] | In vivo | miR-21 | ELISA, RT-PCR | 6 | NF-κB | miR-21 is invovled in the xenon-mediated protective effects in LPS-induced actue kidney injury | 4 |
| Li, 2014 [115] | In vivo | miR-204, miR-211 | RT-PCR | 12 |  | miR-204/miR-211 is involved in the Candidemia-induced kidney dysfunction | 5 |
| Wang, 2009 [116] | In vivo | miR-155 | Microarry, Immunoprecipitation, qRT-PCR | 3 | STAT | mitogen-activated protein kinase phosphatase-1 (MKP-1) adjusts iNOS through modulation of miR-155 | 5 |
| Barnett, 2013 [121] | In vivo | MiR-221 | RT-PCR | 4 | - | dysregualted miRNA is inovled in the inflammatory response | 4 |
| Leelahavanichkul, 2015 [126] | In vivo | miRNA-122 | RT–PCR | 38 | - | miR-122 expression is related to cytokine accumulation | 5 |
| Puimege, 2015 [135] | In vivo | miR-511 | qPCR, ELISA | 4 to 18 | - | miR-511 has anti-inflammatory effect | 5 |
| Acosta-Herrera, 2015 [136] | In vivo | Mir-27a, Mir-103, Mir-17-5p and Mir-130a | microarray, microRNA sequencing | 6 | - | Mir-27a, Mir-103, Mir-17-5p and Mir-130a are deregulated in acute lung injury | 5 |
| Wang, 2013 [138] | In vivo | miRNA-155 | qRT-PCR | 10 | - | miRNA-155 is an mediator in the regulation of inflammation and immunity | 6 |
| Arango, 2014 [146] | In vivo | miR-155 | qRT-PCR | 4 to 6 | - | Downregulation of miR-155 has anti-inflammatory effects | 5 |
| Sari, 2014 [148] | In vivo | miR-150, miR-223, and miR-297 | qRT-PCR | 6 | MyD88/TAK1/IKKb/IjB-a/NF-jB | miR-150, miR-223, and miR-297 are involved in the protective effect of 5,14-HEDGE against inflammation in the rat model of septic shock | 7 |
| Zhang, 2015 [153] | In vivo | miR-146a | RT-qPCR, ELISA, | 6 | - | miR-146a inhibit the release of the inflammatory cytokine TNF-α | 5 |
| Mao, 2015 [58] | In vivo | lncRNAs | qRT-PCR | N/A | LPS-TLR4 | ncRNAs regulate LPS-induced innate immune response in bone marrow-derived macrophages. | 5 |
| Dai, 2016 [145] | In vivo | miR-146a | RT-PCR , Immunohistochemistry | 6 | TLRs/ NF-κB | miR146a limits acute inflammatory response | 4 |
| Lederhuber, 2011 [34] | Humans (Healthy subjects) | miR-146a | RT-PCR | 12 | TLR4 | miR-146a was correlated to neonatal andadult TLR4 signaling | 3 |
| Billeter, 2014 [37] | Humans (Healthy subjects) | miR-155 | ELISA | 7 | - | miRNA-155 intensified the inflammatory response by repressing IL-10 production | 5 |
| Precone, 2013 [9] | Humans (Septic patients) | miR-15,miR-16 | RT-PCR | 80 | - | miR-15,miR-16 were involved in the pro-apoptotic pathway in cirrhotic patients with bacterial infections | 5 |
| How, 2015 [13] | Humans (Septic patients) | let-7a, miR-150 | qRT-PCR | healthy :20, patients : 22 | TLR | let-7a were relevant to the modulation of innate immune response | 5 |
| Goodwin, 2015 [15] | Humans (Septic patients) | miRNA-34a,miR-15a,miR-27a | RT-PCR | septic :62 healthy:32 | - | The plasma levels miRNA-34a,miR-15a,miR-27a were correlated to severe sepsis complicated by shock | 5 |
| Ma, 2013 [16] | Humans (Septic patients) | miR-150 and miR-4772-5p-iso | qRT-PCR | septic:45 healthy: 21 | - | miR-150 and miR-4772-5p-iso were rapid diagnostic assessment of patients on intensive care unit | 4 |
| Yao, 2015 [17] | Humans (Septic patients) | miR-25 | qRT-PCR | patients : 100 | - | miR-25 could be used as biomarker for diagnosis of sepsis and was relevant to anti-oxidation therapy | 4 |
| Wang, 2012 [18] | Humans (Septic patients) | miR-15a, miR-16 | CRP , PCT , qRT-PCR | sepsis patients : 166 , SIRS patients : 32 , healthy :24 | - | Serum miR-15a as a biomarker differentiated between sepsis and SIRS | 5 |
| Wang, 2012 [19] | Humans (Septic patients) | miR-499-5p, miR-122, miR-193b, miR-223 | CRP , PCT , qRT-PCR | septic patients: 166 healthy: 24 | - | miR-499-5p, miR-122, miR-193b, miR-223 were the new biomarkers of sepsis | 5 |
| Wang, 2010 [21] | Humans (Septic patients) | miR-146a, miR-223 | ELISA,qRT-PCR | septic patients: 50, SIRS: 30 healthy: 20 | - | Serum miR-146a and miR-223 were novel biomarkers for sepsis | 5 |
| Wu, 2016 [25] | Humans (Septic patients) | LncRNA-HOTAIR | RT-PCR | 6 | NF-kB | inhibition of LncRNA-HOTAIR had the protective effect on cardiac function of sepsis | 5 |
| Maier, 2008 [36] | Humans (Septic patients) |  | Microarray | 10 | - | chemokines and lipid mediators were associated with systemic inflammation | 3 |
| Liu, 2015 [71] | Humans (Septic patients) | miR-155 | Microarray | sepsis : 60 healthy: 30 | - | miR-155 was a biomarker to determine the severity of sepsis patients | 5 |
| Chen, 2014 [41] | Humans (Septic patients) | miRNAs | RT-PCR,microarray | 24 | - | miRNAs regulated immune response during neonatal sepsis | 5 |
| Wang, 2013 [50] | Humans (Septic patients) | 41 novel miRNAs | qRT-PCR | septic: 94 healthy:24 | - | Six miRNAs were associated with the sepsis outcome | 5 |
| Wang, 2013 [51] | Humans (Septic patients) | miR-150, miR-146a and miR-223 | RT-PCR | septic: 28 | - | miR-146a/U6 was a biomarker to differentiate between sepsis and non-sepsis-SIRS | 5 |
| Shao, 2014 [52] | Humans (Septic patients) | miR-146a | RT-PCR | septic: 226 healthy: 206 | - | miR-146a was involved in the risk of severe sepsis | 5 |
| Wang, 2015 [53] | Humans (Septic patients) | miR-15a/16 | qRT-PCR | septic : 87 | - | miR-15a/16a were the biomarker for the diagnosis and prognosis of sepsis | 5 |
| Wu, 2014 [54] | Humans (Septic patients) | miR-21, miR-125b, miR-132, miR-146a, miR-155 and miR-223 | qRT-PCR | septic : 20 healthy: 15 | - | miR-146a and miR-223 may be used as biomarkers | 5 |
| Zhou, 2015 [57] | Humans (Septic patients) | miR-182, miR-143, miR-145, miR-146a, miR-150, and miR-155 | PCR | septic patients : 32 healthy : 38 | - | miR-182, miR-143, miR-145, miR-146a, miR-150, and miR-155 were related to clinical manifestations and inflammation | 4 |
| van der Heide, 2016 [74] | Humans (Septic patients) | miR-31 | RT-PCR | septic paitents: 23 , healthy patients : 16 | - | miR-31 is a key posttranscriptional regulator in sepsis-related immunosupression | 5 |
| Cui, 2016 [86] | Humans (Septic patients) | miR-130a | ELISA, RT-PCR | 60 | - | miR-130a is associated with thrombocytopenia during severe sepsis | 6 |
| Wang, 2014 [87] | Humans (Septic patients) | miR-122 | RT-PCR | 123 | - | miR-122 is associated with the coagulation disorder in sepsis patients | 6 |
| Vasilescu, 2009 [107] | Humans (Septic patients) | miR-150 | microarray, qRT-PCR, ELISA | sepsis patients: 24 healthy: 32 | - | miR-150 can be used as a marker in the early stage of sepsis | 4 |
| Fredrisksson, 2008 [118] | Humans (Septic patients) | miR-21 | qRT-PCR, Microarray | 17 | - | dysregulation of transcription process cannot compensate for increased damage and proteolysis. | 5 |
| Zhang, 2015 [119] | Humans (Septic patients) | hsa-mir-608 | ELISA | 1268 | - | Hsa-mir-608 can be used as a prognostic biomarker for sepsis in patients with major trauma | 5 |
| Wang, 2012 [122] | Humans (Septic patients) | miR-223, miR-15a, miR-16, miR-122, miR-193, miR-483-5p | qRT-PCR | sepsis patients: 214 healthy: 24 | - | Six serum miRNAs can be used as prognostic markers for sepsis patients | 5 |
| Roderburg, 2013 [123] | Humans (Septic patients) | miR-150 | qRT-PCR | patients: 223 healthy: 76 | - | miR-150 is a prognostic marker in critically ill patients | 5 |
| Tacke, 2014 [124] | Humans (Septic patients) | miR-133a | qRT-PCR | patients: 223 healthy: 76 | - | miR-133a is related to seriousness of diseases | 6 |
| Benz, 2015 [125] | Humans (Septic patients) | miR-223 | qRT-PCR | patients: 221 healthy: 75 | - | miR-223 serum levels should not be used as a biomarker | 6 |
| Wang, 2012 [127] | Humans (Septic patients) | miR-574-5P, miR-297 | microarray, qRT-PCR | 142 | - | serum miR-574-5p is closly related to mortality of spesis patients | 4 |
| Schlosser, 2015 [128] | Humans (Septic patients) | up-, down- regulated RNAs | RT-qPCR | 28 | - | Novel reference controls improve the assessment of disease-related changes in plasma miRNA level | 4 |
| Wang, 2014 [129] | Humans (Septic patients) | Hsa-miR-16, Hsa-miR-15a, Hsa-miR-223, Hsa-miR-122, Hsa-miR-193b | serum proteins measurement | 146 | - | sepsis-related miRNAs can be used as biomakers | 5 |
| Wang, 2014 [130] | Humans (Septic patients) | miR-122 | qRT-PCR | 232 | - | miR-122 is involved in the coagulation disorders in sepsis patients | 5 |
| Yang, 2015 [131] | Humans (Septic patients) | miR-150 | qRT-PCR | 48 | - | miR-150 improve the prognostic efficiency | 5 |
| Wang, 2014 [133] | Humans (Septic patients) | miR-155 | qRT-PCR, ELISA | 60 | - | miR-155 regulate CD4+CD25+ Treg cells proliferation | 4 |
| Ledderose, 2012 [137] | Humans (Septic patients) | miR-124 | qRT-PCR | septic paitents: 24 , healthy patients : 15 | - | miR-124 alleviate anti-inflammatory effects of glucocorticoids | 5 |
| Mohnle, 2015 [154] | Humans (Septic patients) | miR-146a | Flow cytometry analysis, qPCR, | 9 to 14 | - | miR-146a inhibits Th1-cell differentiation | 6 |
| Caserta, 2016 [132] | Humans (Septic patients) | CIR-miRNAs (miR-30d-5p, miR-30a-5p, miR-192-5p,miR-26a-5p, miR-23a-5p, miR-191-5p) | Next-generation sequencing , qRT-PCR | severe SIRS : 23 , severe sepsis : 21 , non-severe SIRS: 21 , non-severe sepsis: 8 , NO SIRS: 16 | - | CIR-miRNAs are inflammatory regulators | 6 |
| Han, 2016 [48] | Humans (Septic patients) | miR-143 | RT–PCR , Serum C-reactive protein (CRP) , procalcitonin (PCT) | sepsis : 103 , SIRS: 95 , healthy: 40 | - | miR-143 is a biomarker to distinguish between sepsis and SIRS | 6 |
| Tili, 2007 [12] | In vitro and in vivo | miR-155.miR-125b | qRT-PCR | 2 to 5 | - | regulation of miR-155 and miR-125b was associated with endotoxin shock | 4 |
| Ho, 2011 [14] | In vitro and in vivo | miR-33 | qRT-PCR | 3 | - | miR-33 regulated the proinflammatory potential in macrophages | 5 |
| Curtis, 2015 [78] | In vitro and in vivo | miR-155 | ELISA | 3 to 14 | - | miR-155 was relevant to innate immune response | 5 |
| Sun, 2012 [45] | In vitro and in vivo | miR-181b | RT-PCR | 0 | NF-κB | miR-181b responsed to proinflammatory stimuli. | 5 |
| Cheng, 2013 [90] | In vitro and in vivo | miR-146 | immunoprecipitation,qRT‐PCR | 4 | - | miR-146 was involved in the pro-inflammatory signalling to affect vascular inflammatory diseases | 5 |
| Moon, 2014 [59] | In vitro and in vivo | miR-15a/16 | ELISA,RT-PCR | 2 to 3 | TLR4 | miR-15a/16a adjusts phagocytosis and bacterial clearance to affect the mortality of septic mice | 5 |
| Zou, 2016 [64] | In vitro and in vivo | miR-146a | miRNA array, qRT-PCR | 3 to 10 | TLR7, MyD88 | Specific miRNAs promote cfB production and AP activation during polymicrobial sepsis | 5 |
| Guan, 2015 [66] | In vitro and in vivo | miR-125b, miR-130a | RT-PCR, CHIP | 15 to 19 | NF-κB/DICER | miR-125b and miR-130a limits the production of TNF-α mRNA | 5 |
| Huang, 2012 [67] | In vitro and in vivo | miRNA-125b | qRT-PCR | 3 to 7 | - | miR-125b regulates proinflammatory responses | 4 |
| McClure, 2014 [72] | In vitro and in vivo | miR-21, miR-181b | RT-PCR, ELISA | 5 | - | miR-21 and miR-181b improve late-sepsis survival | 5 |
| Jiang, 2015 [82] | In vitro and in vivo | miR-19a | qRT-PCR, flow cytometry | SIRS patients: 64 | B cell receptor | miR-19a intensifies the B cell receptor in sepsis | 5 |
| Qi, 2012 [83] | In vitro and in vivo | miR-210 | microRNA arry | 3 | - | miR-210 alleviates LPS-induced production of proinflammatory cytokines | 5 |
| Zhao, 2014 [88] | In vitro and in vivo | miR-218 | qRT-PCR | 10 | Slit2–Robo4 | miR-218 is involved in endothelial inflammation | 6 |
| Rajput, 2016 [91] | In vitro and in vivo | miR-150 | qPCR, ELISA | 4 to 5 | - | miR-150 resolves vascular injury and sepsis by respression of Ang2 generation | 5 |
| Fan, 2014 [93] | In vitro and in vivo | miR-126, miR-125b, miR-34a, and miR-155 | PCR | 3 to 10 | - | miR-126, -125b, -34a, and -155 contribute beneficial effect in sepsis | 5 |
| Wang, 2014 [98] | In vitro and in vivo | miR-27a | qRT-PCR, Microarray | 3 | - | miR-27 adjusts inflammatory responses in sepsis | 5 |
| Ying, 2015 [100] | In vitro and in vivo | miR-127 | qRT-PCR | 3 | JNK | miR-127 is a molecular switch in macrophage development and inflammatory diseases. | 4 |
| Drosatos, 2011 [102] | In vitro and in vivo | MicroRNA | Microarry | 5 | JNK | JNK sigaling limits fatty acid oxidation | 5 |
| Xue, 2015 [109] | In vitro and in vivo | miR-27a | qRT-PCR | 6 | Nrf2 | miR-27a modulation is associated with protection of myocardium | 5 |
| Zhang, 2015 [117] | In vitro and in vivo | ssc-miR-146a-5p, ssc-miR-221-5p, ssc-miR-148b-3p, ssc-miR-215, ssc-miR-192 | qPCR, deep sequencing | 6 | - | miR-146a-5p and miR-221-5p are associated with actue inflammation | 5 |
| El Gazzar, 2010 [139] | In vitro and in vivo | miR-221, miR-579, and miR-125b | Immunoprecipitation, RT-PCR | 3 | TLR4 | miRNAs controls the post-transcriptional process | 5 |
| Banerjee, 2013 [140] | In vitro and in vivo | miR-146a | qPCR, Cytokine assays | 5 | - | miR-146a regulates hyper-inflammation | 5 |
| Zhao, 2014 [147] | In vitro and in vivo | miR-143 | RT-PCR, ELISA , Flow Cytometric Analysis | 3 | - | Downregulation of miR-143 has anti-inflammatory effects | 4 |
| Wang, 2015 [149] | In vitro and in vivo |  | MiRNA microarray, Apoptosis analysis, | 5 to 6 | - | Altering mRNA expressions has the cytoprotective effects | 6 |
| Gao, 2015 [152] | In vitro and in vivo | miR-146a | qPCR, ELISA | 4 to 6 | NF-κB | miR-146a limits sepsis-induced cardiac dysfunction | 5 |
| Sun, 2013 [155] | In vitro and in vivo | miR-124 | RT-PCR, ELISA | 3 | STAT3 | miR-124 regulates LPS-induced cytokine production | 5 |
| Guo, 2016 [157] | In vitro and in vivo | miR-126 | BAL and Cytokine/Chemokine Array, RT-PCR | 3 to 5 | Rac 1 | miR-126 is an important regulator in acute respiratory distress syndrome | 5 |
| Ma, 2016 [46] | In vitro and in vivo | MicroRNA-125b | qPCR, Immunohistochemistry, ELISA | 6 | NF-κB | miR-125b decreases sepsis-induced cardiac dysfunction | 5 |
| Wang, 2016 [47] | In vitro and in vivo | miR-99b | RT–PCR , microRNA array | 3 | - | LPS trigger inflammatory responses by miR-99b medaited machanism | 5 |
| McClure, 2016 [77] | In vitro and in vivo | miR 21 and miR-181b | RT-qPCR , ChIP , | 3 | Stat3 and C/EBPβ | miR21 and miR-181b increase late sepsis immunosuppression and mortality in mice | 5 |
| Androulidaki, 2009 [143] | In vitro and in vivo | let-7e, miR-155, miR-181c and miR125b | RT–PCR | 3 | LPS-TLR | modulation of miRNA is involved in the Akt1-mediated LPS response | 5 |
| Doxaki, 2015 [142] | In vitro and in vivo | miR-146a & miR-155 | quantitative PCR , ChIP | 3 | - | miR-146a and miR-155 contributes Endotoxin tolerance | 5 |
| Schmidt, 2009 [38] | In vivo and healthy subjects | miR-146b, miR-150, miR-342, and let-7g, miR-143 | qRT-PCR | 6 | - | miRNA and its target gene were involved in the LPS-induced inflammation | 5 |
| Li, 2013 [75] | In vivo and septic patients | miR-466l | LC-MS | 4 | - | miR-466l is involved in acute inflammatory responses | 5 |
| Roderburg, 2015 [120] | In vivo and septic patients | miR-122 | qRT-PCR | patients: 223 healthy: 76 mice: 4-12 | - | miR-122 levels can be used as a marker in liver injury and hepatic cell death | 6 |
| Dan, 2015 [60] | In vitro, In vivo and septic patients | miR-181 | RNA-FISH, immunoprecipitation | severe patients :33 | - | miR181 plays an important role in immunoparalysis | 5 |

**Table S1B. Quality assessment of the included studies according to SYRCLE score.**

| First Author, Year [Ref] | | Q1 | Q2 | Q3 | Q4 | Q5 | Q6 | Q7 | Q8 | Q9 | SYRCLE score |
| --- | --- | --- | --- | --- | --- | --- | --- | --- | --- | --- | --- |
| Brudecki, 2013 [6] | | 0 | 0 | 0 | 0 | 0 | 0 | 1 | 1 | 1 | 3 |
| Brudecki, 2013 [7] | | 0 | 0 | 0 | 0 | 0 | 0 | 1 | 1 | 1 | 3 |
| Zheng, 2016 [8] | | 0 | 1 | 0 | 0 | 0 | 1 | 1 | 1 | 1 | 5 |
| Precone, 2013 [9] | | 0 | 1 | 0 | 0 | 0 | 1 | 1 | 1 | 1 | 5 |
| Tili, 2007 [12] | | 0 | 1 | 0 | 0 | 0 | 0 | 1 | 1 | 1 | 4 |
| How, 2015 [13] | | 0 | 1 | 0 | 0 | 0 | 1 | 1 | 1 | 1 | 5 |
| Ho, 2011 [14] | | 0 | 1 | 0 | 0 | 0 | 1 | 1 | 1 | 1 | 5 |
| Goodwin, 2015 [15] | | 0 | 1 | 0 | 0 | 0 | 1 | 1 | 1 | 1 | 5 |
| Ma, 2013 [16] | | 0 | 0 | 0 | 0 | 0 | 1 | 1 | 1 | 1 | 4 |
| Yao, 2015 [17] | | 0 | 0 | 0 | 0 | 0 | 1 | 1 | 1 | 1 | 4 |
| Wang, 2012 [18] | | 0 | 1 | 0 | 0 | 0 | 1 | 1 | 1 | 1 | 5 |
| Wang, 2012 [19] | | 0 | 1 | 0 | 0 | 0 | 1 | 1 | 1 | 1 | 5 |
| Wang, 2010 [21] | | 0 | 1 | 0 | 0 | 0 | 1 | 1 | 1 | 1 | 5 |
| Liu, 2014 [24] | | 0 | 0 | 0 | 0 | 0 | 0 | 1 | 1 | 1 | 3 |
| Wu, 2016 [25] | | 0 | 1 | 0 | 0 | 0 | 1 | 1 | 1 | 1 | 5 |
| Lin, 2015 [26] | | 0 | 0 | 0 | 0 | 0 | 0 | 1 | 1 | 1 | 3 |
| Kanann, 2012 [33] | | 0 | 1 | 0 | 0 | 0 | 0 | 1 | 1 | 1 | 4 |
| Lederhuber, 2011 [34] | | 0 | 0 | 0 | 0 | 0 | 0 | 1 | 1 | 1 | 3 |
| Liu, 2014 [35] | | 0 | 1 | 0 | 0 | 0 | 0 | 1 | 1 | 1 | 4 |
| Maier, 2008 [36] | | 0 | 0 | 0 | 0 | 0 | 0 | 1 | 1 | 1 | 3 |
| Liu, 2015 [71] | | 0 | 1 | 0 | 0 | 0 | 1 | 1 | 1 | 1 | 5 |
| Curtis, 2015 [78] | | 0 | 1 | 0 | 0 | 0 | 1 | 1 | 1 | 1 | 5 |
| Billeter, 2014 [37] | | 0 | 1 | 0 | 0 | 0 | 1 | 1 | 1 | 1 | 5 |
| Schmidt, 2009 [38] | | 0 | 1 | 0 | 0 | 0 | 1 | 1 | 1 | 1 | 5 |
| Wu, 2013 [39] | | 0 | 0 | 0 | 0 | 0 | 1 | 1 | 1 | 1 | 4 |
| Hsieh, 2012 [40] | | 0 | 0 | 0 | 0 | 0 | 1 | 1 | 1 | 1 | 4 |
| Chen, 2014 [41] | | 0 | 1 | 0 | 0 | 0 | 1 | 1 | 1 | 1 | 5 |
| Barnett, 2016 [42] | | 0 | 0 | 0 | 0 | 0 | 1 | 1 | 1 | 1 | 4 |
| Rossato, 2012 [43] | | 0 | 1 | 0 | 0 | 0 | 1 | 1 | 1 | 1 | 5 |
| Sun, 2012 [45] | | 0 | 1 | 0 | 0 | 0 | 1 | 1 | 1 | 1 | 5 |
| Chen, 2013 [90] | | 0 | 1 | 0 | 0 | 0 | 1 | 1 | 1 | 1 | 5 |
| Moore, 2013 [49] | | 1 | 1 | 0 | 0 | 0 | 1 | 1 | 1 | 1 | 6 |
| Wang, 2013 [50] | | 0 | 1 | 0 | 0 | 0 | 1 | 1 | 1 | 1 | 5 |
| Wang, 2013 [51] | | 0 | 1 | 0 | 0 | 0 | 1 | 1 | 1 | 1 | 5 |
| Shao, 2014 [52] | | 0 | 1 | 0 | 0 | 0 | 1 | 1 | 1 | 1 | 5 |
| Wang, 2015 [53] | | 0 | 1 | 0 | 0 | 0 | 1 | 1 | 1 | 1 | 5 |
| Wu, 2014 [54] | | 0 | 1 | 0 | 0 | 0 | 1 | 1 | 1 | 1 | 5 |
| Wang, 2012 [55] | | 0 | 0 | 0 | 0 | 0 | 1 | 1 | 1 | 1 | 4 |
| Zhou, 2015 [57] | | 0 | 1 | 0 | 0 | 0 | 1 | 0 | 1 | 1 | 4 |
| Heyn, 2012 [61] | | 0 | 0 | 0 | 0 | 0 | 0 | 1 | 1 | 1 | 3 |
| Moon, 2014 [59] | | 0 | 1 | 0 | 0 | 0 | 1 | 1 | 1 | 1 | 5 |
| Dan, 2015 [60] | | 0 | 1 | 0 | 0 | 0 | 1 | 1 | 1 | 1 | 5 |
| El Gazzar, 2011 [63] | | 0 | 0 | 0 | 0 | 0 | 0 | 1 | 1 | 1 | 3 |
| Zou, 2016 [64] | | 0 | 1 | 0 | 0 | 0 | 1 | 1 | 1 | 1 | 5 |
| Quinn, 2013 [65] | | 0 | 0 | 0 | 0 | 0 | 0 | 1 | 1 | 1 | 3 |
| Guan, 2015 [66] | | 0 | 1 | 0 | 0 | 0 | 1 | 1 | 1 | 1 | 5 |
| Huang, 2012 [67] | | 0 | 0 | 0 | 0 | 0 | 1 | 1 | 1 | 1 | 4 |
| Du, 2014 [68] | | 0 | 0 | 0 | 0 | 0 | 0 | 1 | 1 | 1 | 3 |
| Li, 2013 [75] |  | 0 | 1 | 0 | 0 | 0 | 1 | 1 | 1 | 1 | 5 |
| Wang, 2014 [76] | | 0 | 0 | 0 | 0 | 0 | 0 | 1 | 1 | 1 | 3 |
| McClure, 2014 [72] | | 0 | 1 | 0 | 0 | 0 | 1 | 1 | 1 | 1 | 5 |
| McClure, 2016 [73] | | 0 | 0 | 0 | 0 | 0 | 1 | 1 | 1 | 1 | 4 |
| van der Heide, 2016 [74] | | 0 | 1 | 0 | 0 | 0 | 1 | 1 | 1 | 1 | 5 |
| Piccinini, 2012 [79] | | 0 | 1 | 0 | 0 | 0 | 1 | 1 | 1 | 1 | 5 |
| Monk, 2010 [80] | | 0 | 0 | 0 | 0 | 0 | 0 | 1 | 1 | 1 | 3 |
| Jiang, 2015 [82] | | 0 | 1 | 0 | 0 | 0 | 1 | 1 | 1 | 1 | 5 |
| Qi, 2012 [83] | | 0 | 1 | 0 | 0 | 0 | 1 | 1 | 1 | 1 | 5 |
| Song, 2015 [84] | | 0 | 0 | 0 | 0 | 0 | 0 | 1 | 1 | 1 | 3 |
| Cui, 2016 [86] | | 1 | 1 | 0 | 0 | 0 | 1 | 1 | 1 | 1 | 6 |
| Wang, 2014 [87] | | 1 | 1 | 0 | 0 | 0 | 1 | 1 | 1 | 1 | 6 |
| Zhao, 2014 [88] | | 1 | 1 | 0 | 0 | 0 | 1 | 1 | 1 | 1 | 6 |
| Chatterjee, 2014 [89] | | 0 | 0 | 0 | 0 | 0 | 0 | 1 | 1 | 1 | 3 |
| Rajput, 2016 [91] | | 0 | 1 | 0 | 0 | 0 | 1 | 1 | 1 | 1 | 5 |
| Wu, 2015 [92] | | 0 | 0 | 0 | 0 | 0 | 0 | 1 | 1 | 1 | 3 |
| Fan, 2014 [93] | | 0 | 0 | 0 | 0 | 0 | 0 | 1 | 1 | 1 | 3 |
| Wang, 2014 [98] | | 0 | 1 | 0 | 0 | 0 | 1 | 1 | 1 | 1 | 5 |
| Liu, 2015 [99] | | 0 | 0 | 0 | 0 | 0 | 0 | 1 | 1 | 1 | 3 |
| Ying, 2015 [100] | | 0 | 0 | 0 | 0 | 0 | 1 | 1 | 1 | 1 | 4 |
| Adyshev, 2013 [101] | | 0 | 0 | 0 | 0 | 0 | 0 | 1 | 1 | 1 | 3 |
| Drosatos, 2011 [102] | | 0 | 1 | 0 | 0 | 0 | 1 | 1 | 1 | 1 | 5 |
| Sun, 2014 [103] | | 0 | 1 | 0 | 0 | 0 | 1 | 1 | 1 | 1 | 5 |
| Ding, 2015 [104] | | 0 | 0 | 0 | 0 | 0 | 1 | 1 | 1 | 1 | 4 |
| Vasilescu, 2009 [107] | | 0 | 0 | 0 | 0 | 0 | 1 | 1 | 1 | 1 | 4 |
| Wang, 2014 [108] | | 0 | 1 | 0 | 0 | 0 | 1 | 1 | 1 | 1 | 5 |
| Xue, 2015 [109] | | 0 | 1 | 0 | 0 | 0 | 1 | 1 | 1 | 1 | 5 |
| Wang, 2016 [110] | | 0 | 1 | 0 | 0 | 0 | 1 | 1 | 1 | 1 | 5 |
| Jia, 2016 [111] | | 0 | 0 | 0 | 0 | 0 | 0 | 1 | 1 | 1 | 3 |
| Risoe, 2011 [112] | | 0 | 1 | 0 | 0 | 0 | 1 | 1 | 1 | 1 | 5 |
| Wang, 2012 [113] | | 1 | 1 | 0 | 0 | 0 | 1 | 1 | 1 | 1 | 6 |
| Jia, 2015 [114] | | 0 | 1 | 0 | 0 | 0 | 1 | 0 | 1 | 1 | 4 |
| Li, 2014 [115] | | 0 | 1 | 0 | 0 | 0 | 1 | 1 | 1 | 1 | 5 |
| Wang, 2009 [116] | | 0 | 1 | 0 | 0 | 0 | 1 | 1 | 1 | 1 | 5 |
| Zhang, 2015 [117] | | 0 | 1 | 0 | 0 | 0 | 1 | 1 | 1 | 1 | 5 |
| Fredrisksson, 2008 [118] | | 0 | 1 | 0 | 0 | 0 | 1 | 1 | 1 | 1 | 5 |
| Zhang, 2015 [119] | | 0 | 1 | 0 | 0 | 0 | 1 | 1 | 1 | 1 | 5 |
| Roderburg, 2015 [120] | | 0 | 1 | 0 | 1 | 0 | 1 | 1 | 1 | 1 | 6 |
| Barnett, 2013 [121] | | 0 | 1 | 0 | 0 | 0 | 1 | 0 | 1 | 1 | 4 |
| Wang, 2012 [122] | | 0 | 1 | 0 | 0 | 0 | 1 | 1 | 1 | 1 | 5 |
| Roderburg, 2013 [123] | | 0 | 1 | 0 | 0 | 0 | 1 | 1 | 1 | 1 | 5 |
| Tacke, 2014 [124] | | 0 | 1 | 0 | 1 | 0 | 1 | 1 | 1 | 1 | 6 |
| Benz, 2015 [125] | | 0 | 1 | 0 | 1 | 0 | 1 | 1 | 1 | 1 | 6 |
| Leelahavanichkul, 2015 [126] | | 0 | 1 | 0 | 0 | 0 | 1 | 1 | 1 | 1 | 5 |
| Wang, 2012 [127] | | 0 | 1 | 0 | 0 | 0 | 1 | 0 | 1 | 1 | 4 |
| Schlosser, 2015 [128] | | 0 | 0 | 0 | 0 | 0 | 1 | 1 | 1 | 1 | 4 |
| Wang, 2014 [129] | | 0 | 1 | 0 | 0 | 0 | 1 | 1 | 1 | 1 | 5 |
| Wang, 2014 [130] | | 0 | 1 | 0 | 0 | 0 | 1 | 1 | 1 | 1 | 5 |
| Yang, 2015 [131] | | 0 | 1 | 0 | 0 | 0 | 1 | 1 | 1 | 1 | 5 |
| Wang, 2014 [133] | | 0 | 0 | 0 | 0 | 0 | 1 | 1 | 1 | 1 | 4 |
| Puimege, 2015 [135] | | 0 | 1 | 0 | 0 | 0 | 1 | 1 | 1 | 1 | 5 |
| Acosta-Herrera, 2015 [136] | | 0 | 1 | 0 | 0 | 0 | 1 | 1 | 1 | 1 | 5 |
| Ledderose, 2012 [137] | | 0 | 1 | 0 | 0 | 0 | 1 | 1 | 1 | 1 | 5 |
| Wang, 2013 [138] | | 1 | 1 | 0 | 0 | 0 | 1 | 1 | 1 | 1 | 6 |
| El Gazzar, 2010 [139] | | 0 | 1 | 0 | 0 | 0 | 1 | 1 | 1 | 1 | 5 |
| Banerjee, 2013 [140] | | 0 | 1 | 0 | 0 | 0 | 1 | 1 | 1 | 1 | 5 |
| Arango, 2014 [146] | | 0 | 1 | 0 | 0 | 0 | 1 | 1 | 1 | 1 | 5 |
| Zhao, 2014 [147] | | 0 | 0 | 0 | 0 | 0 | 1 | 1 | 1 | 1 | 4 |
| Sari, 2014 [148] | | 1 | 1 | 0 | 1 | 0 | 1 | 1 | 1 | 1 | 7 |
| Wang, 2015 [149] | | 0 | 1 | 0 | 1 | 0 | 1 | 1 | 1 | 1 | 6 |
| Shukla, 2015 [151] | | 0 | 0 | 0 | 0 | 0 | 0 | 1 | 1 | 1 | 3 |
| Gao, 2015 [152] | | 0 | 1 | 0 | 0 | 0 | 1 | 1 | 1 | 1 | 5 |
| Zhang, 2015 [153] | | 0 | 1 | 0 | 0 | 0 | 1 | 1 | 1 | 1 | 5 |
| Mohnle, 2015 [154] | | 0 | 1 | 0 | 1 | 0 | 1 | 1 | 1 | 1 | 6 |
| Sun, 2013 [155] | | 0 | 1 | 0 | 0 | 0 | 1 | 1 | 1 | 1 | 5 |
| Guo, 2016 [157] | | 0 | 1 | 0 | 0 | 0 | 1 | 1 | 1 | 1 | 5 |
| Singh, 2016 [27] | | 0 | 0 | 0 | 0 | 0 | 0 | 1 | 1 | 1 | 3 |
| Zhao, 2016 [156] | | 0 | 0 | 0 | 0 | 0 | 0 | 1 | 1 | 1 | 3 |
| Cui, 2014 [28] | | 0 | 0 | 0 | 0 | 0 | 0 | 1 | 1 | 1 | 3 |
| Mao, 2015 [58] | | 0 | 1 | 0 | 0 | 0 | 1 | 1 | 1 | 1 | 5 |
| Ma, 2016 [46] | | 0 | 1 | 0 | 0 | 0 | 1 | 1 | 1 | 1 | 5 |
| Wang, 2016 [47] | | 0 | 1 | 0 | 0 | 0 | 1 | 1 | 1 | 1 | 5 |
| McClure, 2016 [77] | | 0 | 1 | 0 | 0 | 0 | 1 | 0 | 1 | 1 | 4 |
| Caserta, 2016 [132] | | 1 | 0 | 0 | 1 | 0 | 1 | 1 | 1 | 1 | 6 |
| Han, 2016 [48] | | 1 | 0 | 0 | 1 | 0 | 1 | 1 | 1 | 1 | 6 |
| Androulidaki, 2009 [143] | | 0 | 1 | 0 | 0 | 0 | 1 | 1 | 1 | 1 | 5 |
| Nahid, 2009 [141] | | 0 | 0 | 0 | 0 | 0 | 0 | 1 | 1 | 1 | 3 |
| Doxaki, 2015 [142] | | 0 | 1 | 0 | 0 | 0 | 1 | 1 | 1 | 1 | 5 |
| Nahid, 2015 [144] | | 0 | 0 | 0 | 0 | 0 | 0 | 1 | 1 | 1 | 3 |
| Dai, 2016 [145] | | 0 | 1 | 0 | 0 | 0 | 0 | 1 | 1 | 1 | 4 |

Questions used to access the quality of studies (Hooijmans et al, Medical Research Methodology, 2014)

| 1.     Was the allocation sequence adequately generated and applied? | | | | | | |
| --- | --- | --- | --- | --- | --- | --- |
| 2.     Were the groups similar at baseline or were they adjusted for confounders in the analysis? | | | | | | |
| 3.     Was the allocation adequately concealed? | | | | | | |
| 4.     Were the investigators blinded from knowledge which intervention each experimental set received during the experiment? | | | | | | |
| 5.     Were experimental set selected at random for outcome assessment? | | | | | | |
| 6.     Was the outcome assessor blinded? | | | | | | |
| 7.     Were incomplete outcome data adequately addressed? | | | | | | |
| 8.     Are reports of the study free of selective outcome reporting | | | | | | |
| 9.     Was the study apparently free of other problems that could results in high risk of bias? | | | | | | |
|  | | | |  |  |  |
| The questionas were modified from SYRCLE’s risk of bias tool for animal studies; Hooijmans et al, Medical Research Methodology, 2014 | | | | | | |
| One mark was given when the studies complied to the guideline.  Zero score would be given when the studies did not comply | | | |  |  |  |
|  | | | |  |  |  |
|  |  |  |  |  |  |  |
|  |  |  |  |  |  |  |
